# Performance of oxygenation indices and risk scores to predict invasive mechanical ventilation and mortality in COVID-19

**DOI:** 10.1186/s12890-023-02807-8

**Published:** 2024-02-02

**Authors:** Alirio R. Bastidas-Goyes, Eduardo Tuta-Quintero, Maria F. Aguilar, Angélica V. Mora, Hermencia C. Aponte, Jesus M. Villamizar, Susana Galeano, Paola Mejia, Maria Muñoz, Sara Paredes, Doris Pumarejo, Maria Del Mar Barragan

**Affiliations:** 1 School of Medicine, Internal Medicine Department, Universidad de La Sabana, Km 7, Northern highway. Chía, Chía, Cundinamarca 140013 Colombia; 2 Clínica Universidad de La Sabana, Chía, Colombia

**Keywords:** COVID-19, Performance, Mechanical ventilation, Mortality

## Abstract

**Background:**

Information on the performance of oxygenation indices (OIs) and risk scores in patients requiring invasive mechanical ventilation (IMV) is limited. We determine the performance of the OIs and risk scores in hospitalized patients with COVID-19 to predict the requirement of IMV and death at 28 days after admission.

**Methods:**

A retrospective study of diagnostic tests in patients admitted to the emergency department, hospitalization, and intensive care unit diagnosed with COVID-19. The receiver operating characteristic curve (ROC-curve) were built with the OIs and risk scores to predict IMV and mortality.

**Results:**

A total of 1402 subjects entered the final analysis, of whom 19.5% (274/1402) received IMV and 23.0% (323/1402) died at 28 days. The ROC-curve of the delta PaO2/FiO2 ratio for the requirement of IMV and mortality at 28-day was 0.589 (95% CI: 0.546–0.632) and 0.567 (95% CI: 0.526–0.608), respectively. PaO2/FiO2 ≤ 300 shows a ROC curve of 0.669 (95% CI: 0.628–0.711) to predict IMV. PaO2/FiO2 ≤ 300 and 4 C mortality score in mortality at 28 days showed an ROC-curve of 0.624 (95% CI: 0.582–0.667) and 0.706 (95% CI: 0.669–0.742), respectively.

**Conclusion:**

PaO2/FiO2 ≤ 300, 4 C mortality score ≥ 8, SOFA score ≥ 4 y SaO2/FiO2 ≤ 300 were weak predictors of the IMV requirement from admission, and 4 C mortality score ≥ 8 was weak predictors of the mortality from admission in patients with pulmonary involvement by COVID-19.

**Supplementary Information:**

The online version contains supplementary material available at 10.1186/s12890-023-02807-8.

## Introduction

Infection caused by severe acute respiratory syndrome coronavirus type 2 (SARSCoV-2) is responsible for the rapid global spread and current pandemic of coronavirus disease 2019 (COVID-19), which continues to be a threat to public health due to the persistence of reported cases, 6.5 million deaths and the physical sequelae associated with the disease [1, 2]. 80% or more of COVID-19 cases are asymptomatic or mild, however, less than 15% patients with genetic predisposition, comorbidities, or advanced age may develop severe or critical illness with multiple organ failure and acute respiratory distress syndrome, complications causing a high expenditure on medical care resources and a high mortality rate [2, 3].

The main clinical finding in patients with moderate to severe COVID-19 is decreased oxygen saturation values due to diffuse alveolar damage present in acute respiratory distress syndrome and an uncontrolled inflammatory state, [4, 5] generating an imbalance of gas exchange due to destruction of the lung parenchyma, hypercatabolic state and a greater requirement of oxygen pressures for its adequate diffusion through the alveolar capillary membrane [5, 6]. Therefore, patients with severe COVID-19 may require early invasive mechanical ventilation, continuous positive airway pressure, or high-flow nasal cannula, together with strict follow-up consisting of clinical and laboratory tests, including oxygenation indices (OIs) [4, 7, 8].

The use of OIs as predictors of clinical evolution in patients with COVID-19 has already been studied by several authors; Sinatti et al., [9] conducted a cohort study with 150 patients and found that arterial pressure of oxygen/fraction of inspired oxygen (PaO2/FiO2 ratio) can be considered a reliable prognostic biomarker to differentiate severe from mild disease with yield based on the receiver operating characteristic curve (ROC-curve) of 0.838. Xie J et al., [10] showed in 140 patients with pneumonia associated with COVID-19 that supplemental oxygen with a peripheral oxygen saturation (SpO2) lower than 90% was related to sample in more than 60% of the patients, and that an increase in SpO2 improved survival by 8% (HR: 0.92; 95% CI: 0.91 to 0.94; p < 0.001). These results suggest that the evaluation of baseline OI values and their change values in the first hours of clinical follow-up can guide the evolution and prognosis of these patients. The other hand, ROX index (‘*R*espiratory rate-*OX*ygenation’) is the ratio between peripheral blood oxygen saturation (SpO2) to fraction of inspired oxygen (FiO2) and respiratory rate, which has shown promising performance in successful prognosis of invasive and non-invasive oxygen therapy [11–13].

OIs are useful markers for the prediction of complications and mortality in patients with lung involvement, including subjects with COVID-19 [4, 9, 10]. Currently, information on the performance and discriminatory capacity of OIs in patients requiring invasive ventilatory support and mortality are limited, generating significant variability in clinical practice [8, 14]. Based on this, this study aims to determine the performance of OIs, delta of partial pressure of arterial oxygen to fraction of inspired oxygen (PaO2/FiO2) ratio, delta SpO2/FiO2 ratio, ROX index, Sequential Organ Failure Assessment (SOFA) score, 4 C mortality score and Charlson index in hospitalized patients with COVID-19 to predict invasive mechanical ventilation (IMV) and mortality at 28 days after admission.

## Methods

### Study design

A retrospective study of diagnostic tests was carried out in patients admitted to the emergency department, hospitalized, and admitted to the intensive care unit (ICU) of the Clínica Universidad de La Sabana between March 2020 and March 2022. This study followed STARD guidelines for reporting diagnostic or prognostic accuracy Supplementary Table 1.

### Eligibility criteria

The patients included in this study were over 18 years of age and hospitalized for COVID-19 pneumonia for more than 72 h, confirmed by a positive reverse transcription polymerase chain reaction (RT-PCR) test for SARS-CoV-2, obtained with a nasopharyngeal swab/tracheal aspirate or sputum sample. Patients with a history of congenital heart disease leading to chronic hypoxemia and/or home oxygen therapy for these conditions, those requiring mechanical ventilation within the first 6 h of emergency room admission, and those with a single measurement of arterial blood gases that did not allow for clinical follow-up were excluded. Subjects were selected by simple random sampling from the list of patients seen during the study period.

### Analyzed variables

The variables described were age, sex, days since symptoms start, comorbidities (Charlson Index) and ever-smoking or currently smoking tobacco products based on self-reported, vital signs, state of consciousness, complete blood count, ROX index, SOFA score, 4 C mortality score, arterial blood gas, bicarbonate, base excess, arterial oxygen saturation, lactate dehydrogenase, albumin, creatinine, blood ureic nitrogen, lactate dehydrogenase, glutamic-oxaloacetic transaminase, gamma-glutamyl transferase, troponin, procalcitonin, C-Reactive protein, total bilirubin, ferritin, creatine phosphokinase and chest computer tomographic, these data was obtained from medical records at the time of admission to the hospital. SpO2/FiO2 ratio ≤ 350, PaO2/FiO2 ratio ≤ 300, ROX index ≥ 4.88 were calculated from the first 6 h of admission, between 6 and 12 h, 12 to 24 h, and over 24 h [15–17]. Each of the indices were calculated from oximetry records and arterial blood gas measurements. The duration of hospitalization, vasopressor support in the ICU, IMV requirement (7, 14 and 28 days), and death (7, 14 and 28 days) were evaluated.

The cut-off points used for each risk score were ≥ 4.88 for the ROX index, > 4 for the SOFA score and ≥ 8 for the 4 C mortality score (age, respiratory rate, oxygen saturation, blood urea nitrogen and C-Reactive protein) at 12 h as an indicator of failure [11, 18, 19]. The variables of each risk score are described in Supplementary file 2.

### Sample size

To estimate the sample size, data from the study carried out by Alberdí et al., [20] that evaluated SpO2/FiO2 ratio and ROX index, where a sensitivity of 26.8%, specificity of 89.5% for these indices and an expected mortality of 35.3% are reported. For a confidence level of 95% and a precision of 10%, a minimum of 856 patients is required [21].

### Statistical analysis

Data was fully collected and compiled using a secure server (Research Electronic Data Capture, REDCap software) and later analyzed in the SPSS (Statistical Package for the Social Sciences, Chicago, IL) for Windows version 25 [22, 23]. The data were obtained from the medical records of the patients included in the study, and the collection of information was carried out by at least two researchers to reduce the risk of errors in data entry. Quantitative variables were summarized in means and standard deviations if their distribution was normal, or median and interquartile range if their distribution was not normal. The qualitative variables were summarized in frequencies and percentages. A bivariate analysis was performed comparing the sociodemographic variables, comorbidities, laboratory tests, OIs and risk scores with the live and dead outcomes because it provides a good characterization of this population. Differences (delta) were calculated between the SpO2/FiO2 ratio, the PaO2/FiO2 ratio on admission, with those obtained in the first 6 h, between 6 and 12 h, between 12 and 24 h, and over 24 h. For missing data, a weighted median was performed for quantitative variables and logistic regression for qualitative variables [24].

OI (PaO2 mmHg < = 60, SaO2% <= 90, PaO2/FiO2 ≤ 300, SpO2/FiO2 ≤ 350, ΔPaO2/FiO2 ratio, ΔSaO2/FiO2 ratio) of the first 6 h of admission, Charlson index, ROX index of the first 6 h of admission, 4 C mortality score, and SOFA score were calculated to construct the ROC curve with the results of IMV requirement and mortality at 7, 14, and 28 days. The sensitivity, specificity, positive predictive value (PPV), negative predictive value (NPV), positive likelihood ratio (LR+), negative likelihood ratio (LR-) with their respective 95% confidence intervals were calculated. The cut-off points for the calculated deltas were obtained through the Youden index. A comparison was made between the different ROC-curves obtained, using the DeLong test [25]. A p value adjusted by Bonferroni is considered for the comparison of the different OI and risk scores less than < 0.006.

### Ethical considerations

This study was approved by the Ethics Committee of the Clínica Universidad de La Sabana (approval number: 20,220,602), considering it as risk-free research according to resolution 8430 of 1993, and respecting the protection of personal data according to the habeas data law 1266 of 2008.

### Patient and public involvement

Patients were not involved in the development of the research question, design, recruitment, or intervention burden assessed; no patient advisors were required, and data were analyzed anonymously. The results will be disseminated to the scientific community in academic writing.

## Results

### General characteristics of the population, chromobilities and symptoms

**Fig. 1:**
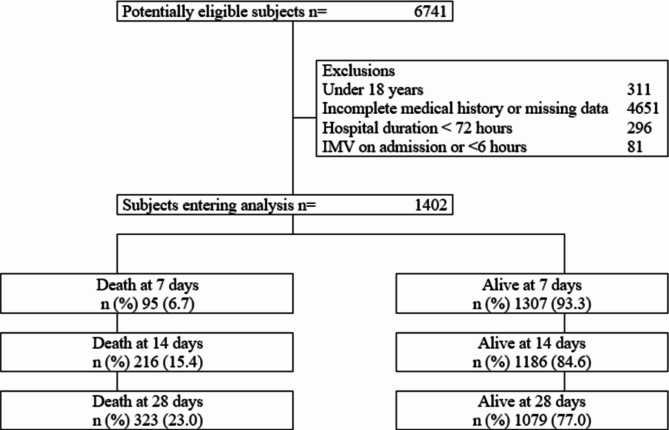
Flow chart of the study. *Notes*: MV, mechanical ventilatory; IMV, invasive mechanical ventilatory

A total of 1402 subjects entered the final analysis, of whom 19.5% (274/1402) received IMV and 23.0% (323/1402) died at 28 days. The 7-day mortality rate was 6.7% (95/1402), and it increased to 15.4% (216/1402) at 14 days. In Fig. 1 the entry flow of subjects to the study is shown. In the general population, the mean age was 59.9 years (SD 16.19), the male sex represented 63.3% (888/1402) and the duration from the onset of symptoms was 7.3 days (SD: 13.26). In the deceased population, 46.4% (150/323) had systemic arterial hypertension compared to 34.5% (372/1079) of the surviving patients (p < 0.001). Cough and crackles occurred in 47.7% (154/323) and 38.4% (124/323) of the patients who died, respectively. The general characteristics are described in Table 1.

**Table 1:** Demographic characteristics, medical history, and risk scores

		Total population n = 1402	Death n = 323	Alive n = 1079	p value	
Age years, mean(sd)		59,9 (16,19)	69.0 (14,22)	57,2 (15,77)	< 0,001	
Male gender, n (%)		888 (63,3)	212 (65,6)	676 (62,7)	0,329	
Days since symptoms start, mean(sd)		7,3 (13,26)	6,2 (5,44)	7,6 (14,8)	0,009	
Comorbidities, n(%)						
Systemic arterial hypertension		522 (37,2)	150 (46.4)	372 (34.5)	< 0.001	
Smoking		542 (38,7)	157 (48,6)	385 (35,7)	< 0.001	
Myocardial infarction		42 (3)	15 (4,6)	27 (2,5)	0.048	
Heart failure		50 (3,6)	22 (6,8)	28 (2,6)	< 0.001	
Peripheral vascular disease		10 (0,7)	2 (0,6)	8 (0,7)	< 0.001	
Cerebrovascular disease		33 (2,4)	18 (5,6)	15 (1,4)	< 0.001	
Chronic lung disease		102 (7,3)	41 (12,7)	61 (5,7)	< 0,001	
Diabetes		218 (15,5)	61 (18,9)	157 (14,6)	0,059	
Chronic kidney disease		75 (5,3)	29 (9)	46 (4,3)	< 0,001	
Charlson index, mean (sd)		2,3 (2,18)	3,6 (2,45)	2 (1,94)	< 0.001	
4 C mortality score *, mean (sd)		8,7 (3,75)	10,6 (3,63)	8 (3,58)	< 0.001	
SOFA score *, mean (sd)		3,6 (2,28)	4,4 (2,5)	3,4 (2,14)	< 0.001	

*Notes*: sd: standard deviation, ROX: Respiratory rate-OXygenation index, SOFA: Sequential Organ Failure Assessment, *: measurement at hospital admission

### Laboratory tests and diagnostic images

The mean pH was 7.43 (SD: 0.07) in the patients who had a fatal outcome versus 7.45 (SD: 0.06) in the survivors (p < 0.001). In the population that died, the average C-reactive protein was 146.4 (SD: 115.74) compared to 121.5 (SD: 107.42) in living patients (p < 0.001). Laboratory tests and diagnostic images are described in Table 2.

**Table 2:** Laboratory tests and diagnostic images

		Total population n = 1402	Death n = 323	Alive n = 1079	p value
Ph, mean(sd)		7.45 (0.06)	7.43 (0.07)	7.45 (0.06)	< 0.001
PaO2 (mmHg), mean(sd)		69 (24.02)	68.3 (24.25)	69.2 (23.96)	0.580
PaCO2(mmHg), mean(sd)		31.7 (6.74)	31.9 (8.35)	31.6 (6.18)	0.859
HCO3 (mE q/L), mean(sd)		22.2 (4.22)	21.7 (4.89)	22.3 (3.99)	< 0.001
BE (mE q/L), mean(sd)		-1.2 (4.05)	-1.9 (4.75)	-1 (3.8)	0.002
Lactate (mmol/L), mean(sd)		1.9 (7.56)	3 (15.72)	1.5 (0.92)	0.089
SaO2 (%), mean(sd)		90.6 (9.76)	89.9 (11.26)	90.8 (9.27)	0.196
Leukocytes, mean(sd)		9537.6 (6981.9)	10410.1 (12053.09)	9275.4 (4420.95)	0.097
Lymphocytes, mean(sd)		1145.7 (950.82)	985.9 (904.55)	1193.6 (959.49)	< 0.001
Neutrophils, mean(sd)		7794.1 (5876.31)	8202.8 (4584.64)	7671.4 (6208.4)	0.094
Hemoglobine, mean(sd)		14.2 (2.44)	13.5 (2.83)	14.4 (2.27)	< 0.001
Hematocrit, mean(sd)		42 (7.68)	40.7 (8.45)	42.5 (7.38)	< 0.001
Plateles, mean(sd)		246220.1 (100052.78)	231,270 (104610.61)	250710.8 (98249.81)	< 0.001
Albumin, mean(sd)		3 (0.62)	2.9 (0.6)	3.1 (0.64)	< 0.001
Creatinine, mean(sd)		1.5 (3.57)	1.8 (2.64)	1.4 (3.8)	0.032
blood ureic nitrogen, mean(sd)		22.1 (15.79)	29.1 (22.25)	20 (12.38)	< 0.001
D dimer, mean(sd)		1827.2 (4861.18)	2811.6 (7119.7)	1536.7 (3914.08)	0.002
LDH, mean(sd)		430.8 (252.17)	506.5 (309.53)	407.9 (227.34)	< 0.001
GOT, mean(sd)		53.7 (55.81)	57 (45.96)	52.6 (58.52)	0.158
GGT, mean(sd)		54.1 (63.57)	48.7 (49.79)	55.7 (67.24)	0.042
Troponin, mean(sd)		37 (101.94)	71.2 (154.89)	26.7 (76.66)	< 0.001
Procalcitonin, mean(sd)		2.1 (8.03)	2.9 (10.1)	1.7 (6.76)	0.047
CRP, mean(sd)		127.3 (109.87)	146.4 (115.74)	121.5 (107.42)	< 0.001
Total bilirubin, mean(sd)		0.8 (2.74)	1.2 (5.51)	0.7 (0.46)	0.093
Ferritin, mean(sd)		1322.4 (2107.67)	1552.8 (2139.67)	1246.3 (2093.1)	0.022
CPK, mean(sd)		453.1 (677.2)	574.1 (835.19)	358.7 (508.47)	< 0.001
Interstitial opacity on CT, mean(sd)		637 (45.44)	148 (45.8)	489 (45.3)	0.874
Alveolar opacity on CT, mean(sd)		446 (31.81)	115 (35.6)	331 (30.7)	0.095
Ground-glass opacity on CT, mean(sd)		556 (39.66)	143 (44.3)	413 (38.3)	0.053

*Notes*: sd: standard deviation, PaO2: arterial oxygen pressure, PaCO2: arterial carbon dioxide pressure, HCO3: bicarbonate, BE: base excess, SaO2: arterial oxygen saturation, LDH: lactate dehydrogenase, GOT: glutamic-oxaloacetic transaminase, GGT: gamma-glutamyl transferase, CRP: C Reactive protein, CPK: Creatine phosphokinase, CT: computer tomography

### Delta in OIs and ROX index in IMV and mortality at 7–28 days

At 28 days, the change in PaO2/FiO2 ratio from 12 to 24 h was − 38.12 (SD: 125.36) in patients with IMV and − 2.16 (SD: 105.44) in patients without mechanical ventilation (p < 0.001) Supplementary Table 3. Additionally, at 28 days, the change in PaO2/FiO2 ratio from 12 to 24 h was − 33.86 (SD: 118.82) in deceased patients and − 1.01 (SD: 112.59) in surviving patients (p < 0.001) Supplementary Table 4.

### Performance of OIs and risk scores in IMV and mortality at 7–14 days

The ROC-curve of the delta PaO2/FiO2 ratio for the requirement of IMV and mortality at 7-days was 0.585 (95% CI: 0.538–0.631) and 0.519 (95% CI: 0.444–0.594), respectively Supplementary Table 5. PaO2/FiO2 ≤ 300 and 4 C mortality score in mortality at 14 days showed an ROC-curve of 0.683 (95% CI: 0.641–0.725) and 0.637 (95% CI: 0.599–0.675), respectively.

### Performance of OIs and risk scores in IMV and mortality at 28 days

The ROC-curve of the delta PaO2/FiO2 ratio for the requirement of IMV and mortality at 28-day was 0.589 (95% CI: 0.546–0.632) and 0.567 (95% CI: 0.526–0.608), respectively Table 3. PaO2/FiO2 ≤ 300 shows a ROC curve of 0.669 (95% CI: 0.628–0.711) to predict IMV. PaO2/FiO2 ≤ 300 and 4 C mortality score in mortality at 28 days showed an ROC-curve of 0.624 (95% CI: 0.582–0.667) and 0.706 (95% CI: 0.669–0.742), respectively.

**Table 3:** Performance of invasive mechanical ventilation and mortality at 28-day

	S (IC 95%)	Sp(IC 95%)	PPV (CI 95%)	NPV (CI 95%)	LR+ (CI 95%)	LR- (CI 95%)	ROC(IC95%)	p value
Invasive Mechanical Ventilation								
PaO2 mmHg < = 60	55.3 (52.7–57.9)	36.8 (34.2–39.3)	44.7 (17.9–15.8)	76.8 (74.6–79)	1.21 (1.082–1.364)	0.88 (0.779–0.983)	0.536 (0.496–0.576)	0.085
SaO2% <= 90	61.4 (58.8–64)	31 (28.5–33.4)	17.5 (15.5–19.6)	77 (74.8-0)	1.25 (1.123–1.384)	0.89 (0.801–0.987)	0.545 (0.505–0.585)	0.032
PaO2/FiO2 ≤ 300	13.9 (12.1–15.8)	71.5 (69.1–73.9)	10.8 (9.2–12.5)	77 (74.7–79.2)	1.2 (0.884–1.641)	0.49 (0.358–0.665)	0.669 (0.628–0.711)	< 0.001
SpO2/FiO2 ≤ 350	54.7 (52-57.3)	25.5 (23.2–27.9)	15.1 (13.2–17)	69.9 (67.5–72.3)	1.77 (1.582–1.989)	0.73 (0.655–0.824)	0.629 (0.588–0.67)	< 0.001
ROX index ≥ 4.8	89.4 (87.8–91.1)	2.5 (1.7–3.4)	18.1 (16.1–20.1)	50 (47.3–52.7)	4.15 (3.978–4.331)	0.92 (0.88–0.958)	0.608 (0.565–0.65)	< 0.001
ΔPaO2/FiO2 ratio	83 (81–85)	5.4 (4.2–6.6)	17.7 (15.7–19.7)	56.3 (53.7–59)	3.16 (2.992–3.348)	0.88 (0.877–0.928)	0.612 (0.573–0.651)	< 0.001
ΔSaO2/FiO2 ratio	90.5 (88.9–92.2)	4.8 (3.6-6)	20.8 (18.6–23.1)	64.8 (62.1–67.5)	1.96 (1.884–2.047)	0.95 (0.912–0.992)	0.589 (0.546–0.632)	< 0.001
SOFA score ≥ 4	55.1 (51.6–58.7)	76.6 (73.6–79.6)	42 (38.5–45.5)	84.7 (82.2–87.3)	2.36 (2.039–2.724)	0.59 (0.507–0.677)	0.633 (0.595–0.671)	< 0.001
4C score ≥ 8	72.1 (69.1–75.1)	48.7 (45.4–52)	29.8 (26.7–32.8)	85.2 (82.9–87.6)	1.4 (1.301–1.515)	0.57 (0.532–0.62)	0.64 (0.602–0.677)	< 0.001
Charlson index ≥ 3	48.5 (45.9–51.1)	61.5 (59-64.1)	23.5 (21.2–25.7)	83.1 (81.2–85.1)	1.26 (1.158–1.374)	0.84 (0.768–0.911)	0.563 (0.524–0.602)	0.003
Mortality								
PaO2 mmHg < = 60	59.2 (56.6–61.8)	37.6 (35.1–40.2)	22.1 (19.9–24.3)	75.5 (73.2–77.8)	1.08 (0.979–1.157)	0.95 (0.856–1.052)	0.515 (0.474–0.557)	0.449
SaO2% <= 90	66 (63.5–68.5)	32 (29.5–34.5)	22.3 (20.1–24.5)	76.1 (73.8–78.4)	1.06 (0.986–1.25)	0.97 (0.886–1.063)	0.513 (0.472–0.553)	0.536
PaO2/FiO2 ≤ 300	70.6 (68.2–73)	9.3 (7.8–10.8)	18.9 (18.9–21)	51.3 (48.7–54)	3.17 (2.943–3.411)	0.78 (0.723–0.838)	0.624 (0.582–0.667)	< 0.001
SaO2/FiO2 ≤ 350	58.8 (56.2–61.4)	25.9 (23.6–28.2)	19.1 (17-21.1)	67.9 (65.4–70.4)	1.59 (1.441–1.757)	0.79 (0.718–0.876)	0.581 (0.539–0.623)	< 0.001
ROX index ≥ 4.8	91.6 (90.2–93.1)	2.8 (2-3.7)	21.8 (19.6–24)	53.6 (50.9–56.2)	2.94 (2.836–3.05)	0.94 (0.911–0.977)	0.588 (0.545–0.63)	< 0.001
ΔPaO2/FiO2 ratio	81.1 (79-83.2)	6.5 (5.2–7.8)	20.7 (18.6–22.9)	53.2 (50.5–55.8)	2.92 (2.76–3.086)	0.87 (0.82–0.917)	0.547 (0.507–0.587)	0.021
ΔSaO2/FiO2 ratio	87.9 (86-89.7)	7.3 (5.9–8.8)	20.8 (18.5–23.1)	68.6 (66-71.2)	1.65 (1.574–1.734)	0.95 (0.904–0.995)	0.567 (0.526–0.608)	0.001
SOFA score ≥ 4	58.1 (54.6–61.7)	75.9 (72.8–78.9)	37.2 (33.8–40.7)	88 (85.7–90.4)	2.41 (1.978–2.933)	0.55 (0.453–0.672)	0.633 (0.595–0.67)	< 0.001
4C score ≥ 8	74.9 (72-77.8)	48.2 (44.9–51.5)	25.3 (22.4–28.2)	89.1 (87.1–91.2)	1.45 (1.292–1.619)	0.52 (0.466–0.584)	0.706 (0.669–0.742)	< 0.001
Charlson index ≥ 3	49.8 (47.2–52.4)	61.3 (58.7–63.8)	19.2 (17.2–21.3)	86.8 (85.1–88.6)	1.29 (1.105–1.495)	0.82 (0.705–0.953)	0.672 (0.636–0.709)	< 0.001

*Notes*: S: Sensibility, Sp: specificity, PPV: positive predictive value, NPV: negative predictive value, LR +: positive likelihood ratio, LR-: negative likelihood ratio, CI: confidence intervals, ROC: receiver operating characteristic curve, PaO2: arterial oxygen pressure, SaO2: arterial oxygen saturation, Δ: delta, PaO2/FiO2 ratio: arterial oxygen pressure/inspired fraction of oxygen, SaO2/FiO2 ratio: arterial oxygen saturation in relation to the inspired oxygen fraction, ROX: Respiratory rate-OXygenation index, SOFA: Sequential Organ Failure Assessment

**Fig. 2:**
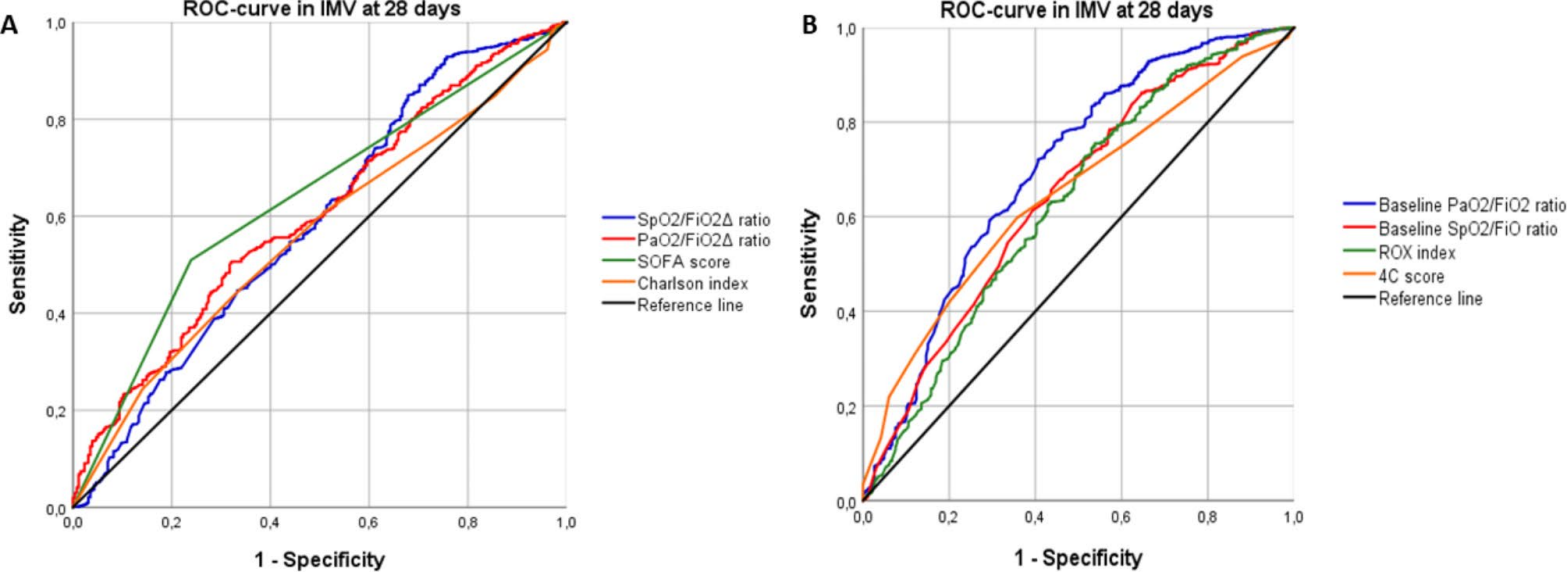
Performance of oxygenation indices and risk scores in invasive mechanical ventilation at 28 days. *Notes*: ROC-curve: receiver operating characteristic curve, IMV: invasive mechanical ventilation, PaO2/FiO2 ratio: arterial oxygen pressure/inspired fraction of oxygen, SpO2/FiO2, arterial oxygen saturation/fraction of inspired oxygen, ROX: Respiratory rate-OXygenation index, SOFA: Sequential Organ Failure Assessment, Δ: delta

**Fig. 3:**
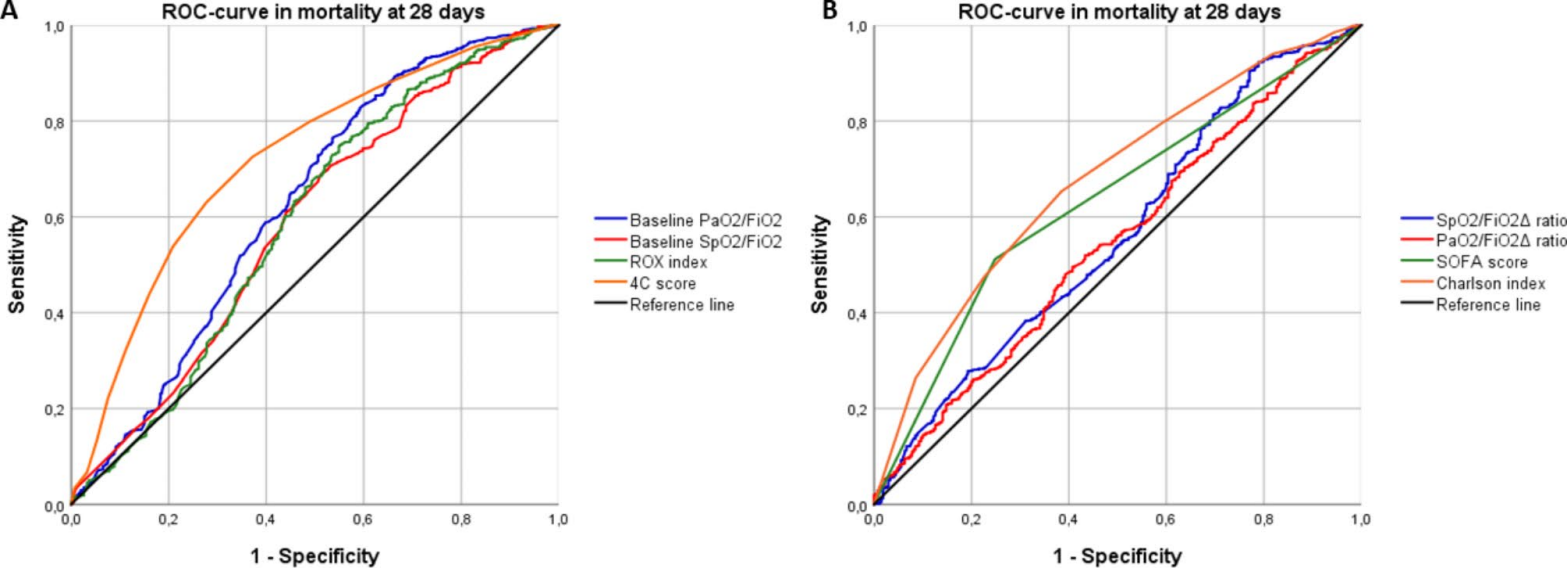
Performance of oxygenation indices and risk scores in mortality at 28 days. *Notes*: ROC-curve: receiver operating characteristic curve, IMV: invasive mechanical ventilation, PaO2/FiO2 ratio: arterial oxygen pressure/inspired fraction of oxygen, SpO2/FiO2, arterial oxygen saturation/fraction of inspired oxygen, ROX: Respiratory rate-OXygenation index, SOFA: Sequential Organ Failure Assessment, Δ: delta

The ROC-curve of the OIs and the risk scores to predict IMV and mortality are shown in the Fig. 2 and Fig. 3.

Analysis with the De Long test showed that at 7, 14 and 28 days the ROC-curves with the best performance for IMV were PaO2/FiO2, 4 C mortality score, SOFA score and SaO2/FiO2, the ROC curves of the rest of the OI evaluated were lower (p < 0.001). At 7 days, no statistically significant differences were found for multiple comparisons between the OI and risk scores assessed for death (p = 0.043). At 14 days, ROC-curves with the best behavior for death were the 4 C mortality score and the Charlson index, the ROC-curves of the rest of the OI and SOFA scores evaluated were lower (p < 0.001). At 28 days, the ROC-curve with the best behavior for death was 4 C mortality score compared to the ROC-curves of the rest of the OI and risk scores evaluated (p < 0.001).

## Discussion

The present study determined the performance of the change in OIs and risk scores in a retrospective study of diagnostic tests of 1402 hospitalized patients with COVID-19, finding that the PaO2/FiO2, 4 C mortality score, SOFA score y SaO2/FiO2 were weak predictors of the IMV requirement from admission, and 4 C mortality score was weak predictors of the mortality from admission. The delta PaO2/FiO2 and the delta SaO2/FiO2 showed inferior performance for IMV and death compared to the other tools evaluated. Characteristics such as age, pathological history, and clinical manifestations occurred more frequently among patients who died from SARS-CoV-2. Our findings suggest that the predictive capacity for the requirement of IMV and mortality is limited. This is attributed to the fact that ROC-curves were independently calculated for each of the OIs and risk indices, which primarily focus on describing respiratory compromise and the reduction in gas exchange across the alveolar-capillary membrane [2, 3, 9, 10]. Therefore, conducting integrated assessments that consider both OIs and risk indices is essential to estimate damage across various organs or systems, as commonly observed in severe pneumonia cases caused by COVID-19.

PaO2/FiO2 reflects the severity of hypoxemia and given its performance, has been correlated with prognosis and hospital mortality in patients with acute respiratory failure due to COVID-19 [26–28]. Direct blood sample collection for gasometric analysis it is the main reference for evaluating the oxygenation status of patients with respiratory diseases, [9] and its baseline value is the one that offers the most information in the evaluation of the patient [9, 27, 28]. In our study, the PaO2/FiO2 ratio showed a weak discriminatory capacity for IMV or death. In addition, the continuous evaluation of oxygenation status through gas analysis constitutes an expensive invasive procedure and was not available at the different levels of care, requiring the continuous study of other measurements that use pulse oximetry to obtain indices such as the SpO2/FiO2 ratio and ROX index, which can reflect the state of hypoxemia in a non-invasive way [29].

Knight et al., [18] developed and validated a 4 C mortality score (Coronavirus Clinical Characterization Consortium) of risk for the prediction of mortality in a derivation cohort with 35.463 patients and validation with 22.361 patients hospitalized for COVID-19. The variables of age, gender, number of comorbidities, respiratory rate, SpO2, state of consciousness, urea nitrogen, and C Reactive protein were included in the score; obtaining a scale of 0 to 21 points and a performance of 0.79 (95% CI: 0.78–0.79) in the derivation cohort and 0.77 (95% CI: 0.76–0.77) in the validation cohort for mortality, similar findings in our study where greater comorbidity measured by Charlson and greater severity by 4 C mortality score were related to greater mortality in patients with COVID-19 [18, 30, 31].

Cattazzo et al., [32] analyzed the efficiency of the ROX index and the SaO2/FiO2 ratio compared to PaO2/FiO2 ratio for the prediction of death or IMV requirement in 456 patients hospitalized in areas other than the ICU due to COVID-19. The results showed an ROC-curve of 0.67 (95% CI: 0.62–0.73; p < 0.001) for PaO2/FiO2 ratio, 0.69 (95% CI: 0.63–0.74; p < 0.001) for the ROX index and 0.66 (95% CI: 0.60–0.72; p < 0.001) for SpO2/FiO2 ratio; similar situation to what we find where the basal values ​​of each of these indices have a weak discriminatory capacity for IMV or death. Baek et al., [33] in patients with COVID-19 and supplemental oxygen with a high-flow cannula found that the change in the ROX index and SaO2/FiO2 ratio was related to early IMV; findings that were corroborated with our results where a drop in SaO2/FiO2 ratio of 150 points was associated with a greater requirement of IMV and death, the change in this variable being the one with the best efficiency for the prediction of complications in patients with SARS-CoV2 infection.

Grasselli et al., [34] described demographic characteristics, comorbidities, and treatments of a cohort of 3,988 critically ill patients with SARS-CoV-2 infection. Hypertension, hypercholesterolemia, heart disease, diabetes, chronic obstructive pulmonary disease, and chronic kidney disease were associated with increased mortality. Our results confirm that deaths from COVID-19 were higher in patients with multiple comorbidities, a consequence of a pre-existing inflammatory state in chronic diseases and greater susceptibility to a cellular immune response and humoral activation, predominantly of tumor necrosis factor and interleukin 6 [35, 36]. This was possibly related to what was observed in most clinical scenarios as described during the pandemic period, in which the presence of other respiratory diseases directly influences the loss of alveolar reserve, promoting greater pulmonary compromise secondary to viral infection [37, 38].

### Limitations

As it was a retrospective study based on medical records, this study may give rise to selection and information biases; However, we implemented measures to minimize bias, such as training the personnel in charge of collecting medical data and constructing the manuscript based on the checklist of items that should be included in the reports of retrospective diagnostic test study Supplementary Table 1. Similarly, being a single center study may limit the extrapolation of the results, despite this, there was a sufficient sample size to support them. Unlike the ROX index and the 4 C mortality score, the SOFA score has an important limitation in its daily application, since it depends on variables that are not always available in medical care centers or hospitals, especially in countries with limited resources, as in our study population [11, 18, 19].

The altitude above sea level where the care center was located can be considered a limiting factor, since exceeding 2.500 m above sea level can alter oxygenation values, as described in previous studies [13, 39]. However, there are large numbers of the world’s population residing at altitudes to which these findings could be applied. The lack of a uniform take in the evaluation of arterial gasses during the evolution of patients with SARS-CoV2 can cause confusion throughout the follow-up, however, each of the analyzes at different periods of time carried out in the study consistently show differences in the OIs evaluated. We consider it pertinent to carry out future studies that corroborate our findings.

## Conclusions

PaO2/FiO2 ≤ 300, 4 C mortality score ≥ 8, SOFA score ≥ 4 y SaO2/FiO2 ≤ 300 were weak predictors of the IMV requirement from admission, and 4 C mortality score ≥ 8 was weak predictors of the mortality from admission in patients with pulmonary involvement by COVID-19. Age, pathological history, and clinical manifestations occurred more frequently among patients who died from SARS-CoV-2.

### Electronic supplementary material

Below is the link to the electronic supplementary material.

**Supplementary Material 1:** Standards for Reporting Diagnostic accuracy studies Checklist

**Supplementary Material 2:** ROX index

**Supplementary Material 3:** Delta in oxygenation indices and ROX index in invasive mechanical ventilation

**Supplementary Material 4:** Delta oxygenation indices and ROX index in mortality

**Supplementary Material 5:** Performance of oxygenation indices and risk scores in invasive mechanical ventilation and mortality at 7-14 days

## Data Availability

The datasets generated during and/or analyzed during the current study are available from the corresponding author on reasonable request.
