# Supplementary material for: Performance of oxygenation indices and risk scores to predict invasive mechanical ventilation and mortality in COVID-19
Source: BMC Pulm Med. 2024 Feb 2;24:68. doi: 10.1186/s12890-023-02807-8 (PMC10835882; doi:10.1186/s12890-023-02807-8)
Supplement: Supplementary file 2 — Supplementary Material 2: ROX index [file 12890_2023_2807_MOESM2_ESM.docx]

**Supplementary table 2a.** ROX index

| SpO₂ | SpO_2_/FiO2  Respiratory Rate |
| --- | --- |
| FiO₂ |  |
| Respiratory rate |  |

**Supplementary table 2b.** SOFA score

| **Score** | **0** | **1** | **2** | **3** | **4** |
| --- | --- | --- | --- | --- | --- |
| *Respiratory* |  |  |  |  |  |
| PaO_2_/FiO_2_, mmHg | > 400 | ≤ 400 | ≤ 300 | ≤ 200 | ≤ 100 |
|  |  |  |  | —with respiratory support— |  |
| *Coagulation* |  |  |  |  |  |
| Platelets × 10^3^/mm^3^ | > 150 | ≤ 150 | ≤ 100 | ≤ 50 | ≤ 20 |
| *Liver* |  |  |  |  |  |
| Bilirubin, mg/dL (μmol/L) | < 1.2 (< 20) | 1.2–1.9 (20–32) | 2.0–5.9 (33–101) | 6.0–11.9 (102–204) | > 12.0 (> 204) |
| *Cardiovascular* |  |  |  |  |  |
| Hypotension | No hypotension | MAP < 70 mmHg | Dopamine ≤ 5 or dobutamine (any dose)* | Dopamine > 5 or epinephrine ≤ 0.1 or norepinephrine ≤ 0.1* | Dopamine > 15 or epinephrine > 0.1 or norepinephrine > 0.1* |
| *Central nervous system* |  |  |  |  |  |
| Glasgow Coma Scale | 15 | 13–14 | 10–12 | 6–9 | < 6 |
| *Renal* |  |  |  |  |  |
| Creatinine, mg/dL (μmol/L) | < 1.2 (< 110) | 1.2–1.9 (110–170) | 2.0–3.4 (171–299) | 3.5–4.9 (300–440) | > 5.0 (> 440) |
| OR urine output |  |  |  | < 500 ml/d | < 200 ml/d |

^*^Adrenergic agents administered for at least one hour (doses given are in mcg/kg/min)

**Supplementary table 2c.** 4C Mortality Score

| **Variable** | **4C Mortality Score** |
| --- | --- |
| Age (years) |  |
| <50 | — |
| 50-59 | +2 |
| 60-69 | +4 |
| 70-79 | +6 |
| ≥80 | +7 |
| Sex at birth |  |
| Female | — |
| Male | +1 |
| No of comorbidities* |  |
| 0 | — |
| 1 | +1 |
| ≥2 | +2 |
| Respiratory rate (breaths/min) |  |
| <20 | — |
| 20-29 | +1 |
| ≥30 | +2 |
| Peripheral oxygen saturation on room air (%) |  |
| ≥92 | — |
| <92 | +2 |
| Glasgow coma scale score |  |
| 15 | — |
| <15 | +2 |
| Urea (mmol/L) |  |
| <7 | — |
| 7-14 | +1 |
| >14 | +3 |
| C reactive protein (mg/L) |  |
| <50 | — |
| 50-99 | +1 |
| ≥100 | +2 |
