# Supplementary material for: Performance of oxygenation indices and risk scores to predict invasive mechanical ventilation and mortality in COVID-19
Source: BMC Pulm Med. 2024 Feb 2;24:68. doi: 10.1186/s12890-023-02807-8 (PMC10835882; doi:10.1186/s12890-023-02807-8)
Supplement: Supplementary file 3 — Supplementary Material 3: Delta in oxygenation indices and ROX index in invasive mechanical ventilation [file 12890_2023_2807_MOESM3_ESM.docx]

**Supplementary table 3.** Delta in oxygenation indices and ROX index in invasive mechanical ventilation.

|  | | Total population n= 1402 | IMV at 7 days n= 274 | No IMV n= 1128 | p value |  |
| --- | --- | --- | --- | --- | --- | --- |
|  |  |  |  |  |  |  |
| Δ SpO2/FiO2 ratio 6 hours | | -41.15 (83.96) | -65.57 (107.45) | -35.59 (76.68) | <0.001 |  |
| Δ SpO2/FiO2 ratio 6 to 12 hours | | -49.63 (90.76) | -65.18 (105.09) | -46.35 (87.3) | 0.003 |  |
| Δ SpO2/FiO2 ratio 12 to 24 hours | | -37.55 (82.43) | -86.85 (110.22) | -31.9 (76.91) | <0.001 |  |
| Δ SpO2/FiO2 ratio greater 24 horas | | -37.55 (82.43) | -86.85 (110.22) | -31.9 (76.91) | <0.001 |  |
|  |  |  |  |  |  |  |
|  |  | Total population n= 1402 | IMV at 14 days n= 274 | No IMV n= 1128 | p value |  |
|  |  |  |  |  |  |  |
| Δ SpO2/FiO2 ratio 6 hours | | -41.15 (83.96) | -61.49 (112.01) | -35.7 (73.89) | <0.001 |  |
| Δ SpO2/FiO2 ratio 6 to 12 hours | | -49.63 (90.76) | -79.36 (104.72) | -41.47 (84.95) | <0.001 |  |
| Δ SpO2/FiO2 ratio 12 to 24 hours | | -37.55 (82.43) | -72.9 (109.48) | -32.21 (76.41) | <0.001 |  |
| Δ SpO2/FiO2 ratio greater 24 horas | | -37.55 (82.43) | -72.9 (109.48) | -32.21 (76.41) | <0.001 |  |
|  |  |  |  |  |  |  |
|  |  | Total population n= 1402 | IMV at 28 days n= 274 | No IMV n= 1128 | p value |  |
|  |  |  |  |  |  |  |
| Δ SpO2/FiO2 ratio 6 hours | | -41.15 (83.96) | -60.65 (110.99) | -35.71 (73.94) | <0.001 |  |
| Δ SpO2/FiO2 ratio 6 to 12 hours | | -49.63 (90.76) | -71.31 (109.13) | -43.15 (83.66) | <0.001 |  |
| Δ SpO2/FiO2 ratio 12 to 24 hours | | -37.55 (82.43) | -69.53 (107.57) | -32.42 (76.69) | <0.001 |  |
| Δ SpO2/FiO2 ratio greater 24 horas | | -37.55 (82.43) | -69.53 (107.57) | -32.42 (76.69) | <0.001 |  |
|  | |  |  |  |  |  |
|  |  | Total population n= 1402 | IMV at 7 days n= 274 | No IMV n= 1128 | p value |  |
|  |  |  |  |  |  |  |
| Δ ROX at 6 hours | | -1.61 (4.85) | -3.07 (5.84) | -1.28 (4.54) | <0.001 |  |
| Δ ROX 6 to 12 hours | | -2.04 (5.65) | -2.89 (6.61) | -1.86 (5.42) | 0.011 |  |
| Δ ROX 12 to 24 hours | | -1.4 (5.17) | -3.73 (5.84) | -1.14 (5.03) | <0.001 |  |
| Δ ROX greater 24 hours | | -1.62 (5.85) | -5.31 (5.97) | -1.16 (5.7) | <0.001 |  |
|  |  |  |  |  |  |  |
|  |  | Total population n= 1402 | IMV at 14 days n= 274 | No IMV n= 1128 | p value |  |
|  |  |  |  |  |  |  |
| Δ ROX at 6 hours | | -1.61 (4.85) | -2.82 (5.95) | -1.29 (4.46) | <0.001 |  |
| Δ ROX 6 to 12 hours | | -2.04 (5.65) | -3.56 (6.38) | -1.63 (5.37) | <0.001 |  |
| Δ ROX 12 to 24 hours | | -1.4 (5.17) | -3.11 (6.1) | -1.15 (4.98) | <0.001 |  |
| Δ ROX greater 24 hours | | -1.62 (5.85) | -4.39 (6.31) | -1.1 (5.65) | <0.001 |  |
|  |  |  |  |  |  |  |
|  |  | Total population n= 1402 | IMV at 28 days n= 274 | No IMV n= 1128 | p value |  |
|  |  |  |  |  |  |  |
| Δ ROX at 6 hours | | -1.61 (4.85) | -2.77 (5.89) | -1.29 (4.48) | <0.001 |  |
| Δ ROX 6 to 12 hours | | -2.04 (5.65) | -3.28 (6.45) | -1.67 (5.34) | <0.001 |  |
| Δ ROX 12 to 24 hours | | -1.4 (5.17) | -3.07 (5.96) | -1.14 (4.99) | <0.001 |  |
| Δ ROX greater 24 hours | | -1.62 (5.85) | -4.16 (6.03) | -1.08 (5.7) | <0.001 |  |
|  |  |  |  |  |  |  |
|  |  | Total population n= 1402 | IMV at 7 days n= 274 | No IMV n= 1128 | p value |  |
|  |  |  |  |  |  |  |
| Δ PaO2/FiO2 ratio at 6 hours | | -25.21 (114.2) | -48.85 (96.15) | -6.35 (124.07) | <0.001 |  |
| Δ PaO2/FiO2 ratio 6 a 12 hours | | -35.83 (105.5) | -58.8 (107.43) | -26.47 (103.58) | <0.001 |  |
| Δ PaO2/FiO2 ratio 12 a 24 hours | | -8.38 (114.53) | -37.07 (121.55) | -4.59 (110.31) | <0.001 |  |
| Δ PaO2/FiO2 ratio greater 24 hours | | 174.52 (112.06) | 82.22 (67.94) | 179.35 (116.76) | <0.001 |  |
|  |  |  |  |  |  |  |
|  |  | Total population n= 1402 | IMV at 14 days n= 274 | No IMV n= 1128 | p value |  |
|  |  |  |  |  |  |  |
| Δ PaO2/FiO2 ratio at 6 hours | | -25.21 (114.2) | -51.3 (99.43) | -0.02 (122.21) | <0.001 |  |
| Δ PaO2/FiO2 ratio 6 a 12 hours | | -35.83 (105.5) | -61.8 (110.89) | -23.1 (100.7) | <0.001 |  |
| Δ PaO2/FiO2 ratio 12 a 24 hours | | -8.38 (114.53) | -32.24 (125.41) | -3.87 (106.01) | <0.001 |  |
| Δ PaO2/FiO2 ratio greater 24 hours | | 174.52 (112.06) | 91.53 (76.83) | 180.08 (114.3) | <0.001 |  |
|  |  |  |  |  |  |  |
|  |  | Total population n= 1402 | IMV at 28 days n= 274 | No IMV n= 1128 | p value |  |
|  |  |  |  |  |  |  |
| Δ PaO2/FiO2 ratio at 6 hours | | -25.21 (114.2) | -53.18 (99.14) | 3.09 (121.83) | <0.001 |  |
| Δ PaO2/FiO2 ratio 6 a 12 hours | | -35.83 (105.5) | -64.44 (110.68) | -21.23 (100.01) | <0.001 |  |
| Δ PaO2/FiO2 ratio 12 a 24 hours | | -8.38 (114.53) | -38.12 (125.36) | -2.16 (105.44) | <0.001 |  |
| Δ PaO2/FiO2 ratio greater 24 hours | | 174.52 (112.06) | 94.3 (79.02) | 180.38 (114.7) | <0.001 |  |

Notes: Δ: delta; SpO2/FiO2 ratio: arterial oxygen saturation in relation to the inspired oxygen fraction; ROX: Respiratory rate-OXygenation index; PaO2/FiO2 ratio: arterial oxygen pressure/inspired fraction of oxygen.
