# Supplementary material for: Performance of oxygenation indices and risk scores to predict invasive mechanical ventilation and mortality in COVID-19
Source: BMC Pulm Med. 2024 Feb 2;24:68. doi: 10.1186/s12890-023-02807-8 (PMC10835882; doi:10.1186/s12890-023-02807-8)
Supplement: Supplementary file 4 — Supplementary Material 4: Delta oxygenation indices and ROX index in mortality [file 12890_2023_2807_MOESM4_ESM.docx]

**Supplementary table 4.** Delta oxygenation indices and ROX index in mortality.

|  |  | Total population n= 1402 | Death at 7 days n= 323 | Alive n= 1079 | p value |  |
| --- | --- | --- | --- | --- | --- | --- |
|  |  |  |  |  |  |  |
| Δ SpO2/FiO2 ratio at 6 hours | | -41.15 (83.96) | -77.75 (104.68) | -38.3 (81.57) | <0.001 |  |
| Δ SpO2/FiO2 ratio 6 to 12 hours | | -49.63 (90.76) | -51.39 (92.42) | -49.49 (90.78) | 0.742 |  |
| Δ SpO2/FiO2 ratio 12 to 24 hours | | -37.55 (82.43) | -42.62 (47.67) | -37.24 (84.11) | 0.145 |  |
| Δ SpO2/FiO2 ratio greater 24 horas | | -37.55 (82.43) | -42.62 (47.67) | -37.24 (84.11) | 0.145 |  |
|  |  | Total population n= 1402 | Death at 14 days n= 323 | Alive n= 1079 | p value |  |
|  |  |  |  |  |  |  |
| Δ SpO2/FiO2 ratio at 6 hours | | -41.15 (83.96) | -68.59 (106.14) | -36.52 (78.81) | <0.001 |  |
| Δ SpO2/FiO2 ratio 6 to 12 hours | | -49.63 (90.76) | -62.28 (100.76) | -46.57 (88.1) | 0.012 |  |
| Δ SpO2/FiO2 ratio 12 to 24 hours | | -37.55 (82.43) | -72.9 (98.13) | -32.21 (78.64) | <0.001 |  |
| Δ SpO2/FiO2 ratio greater 24 horas | | -37.55 (82.43) | -71.38 (98.13) | -32.28 (78.64) | <0.001 |  |
|  |  | Total population n= 1402 | Death at 28 days n= 323 | Alive n= 1079 | p value |  |
|  |  |  |  |  |  |  |
| Δ SpO2/FiO2 ratio at 6 hours | | -41.15 (83.96) | -56.43 (103.25) | -36.4 (76.51) | 0.001 |  |
| Δ SpO2/FiO2 ratio 6 to 12 hours | | -49.63 (90.76) | -61.87 (105.95) | -45.53 (84.92) | 0.011 |  |
| Δ SpO2/FiO2 ratio 12 to 24 hours | | -37.55 (82.43) | -68.99 (97.22) | -29.05 (75.99) | <0.001 |  |
| Δ SpO2/FiO2 ratio greater 24 horas | | -37.55 (82.43) | -68.99 (97.22) | -29.05 (75.99) | <0.001 |  |
|  |  | Total population n= 1402 | Death at 7 days n= 323 | Alive n= 1079 | p value |  |
|  |  |  |  |  |  |  |
| Δ ROX at 6 hours | | -1.61 (4.85) | -2.65 (5.66) | -1.53 (4.78) | 0.001 |  |
| Δ ROX 6 to 12 hours | | -2.04 (5.65) | -1.32 (5.89) | -2.1 (5.63) | 0.030 |  |
| Δ ROX 12 to 24 hours | | -1.4 (5.17) | -2.04 (3.54) | -1.37 (5.25) | 0.008 |  |
| Δ ROX greater a 24 hours | | -1.62 (5.85) | -3.96 (1.88) | -1.53 (5.94) | <0.001 |  |
|  |  | Total population n= 1402 | Death at 14 days n= 323 | Alive n= 1079 | p value |  |
|  |  |  |  |  |  |  |
| Δ ROX at 6 hours | | -1.61 (4.85) | -2.63 (5.67) | -1.44 (4.68) | 0.223 |  |
| Δ ROX 6 to 12 hours | | -2.04 (5.65) | -2.09 (6.91) | -2.03 (5.31) | 0.884 |  |
| Δ ROX 12 to 24 hours | | -1.4 (5.17) | -3.03 (5.18) | -1.15 (5.13) | <0.001 |  |
| Δ ROX greater a 24 hours | | -1.62 (5.85) | -6.83 (5.96) | -0.85 (5.46) | <0.001 |  |
|  |  | Total population n= 1402 | Death at 28 days n= 323 | Alive n= 1079 | p value |  |
|  |  |  |  |  |  |  |
| Δ ROX at 6 hours | | -1.61 (4.85) | -2.25 (5.57) | -1.42 (4.6) | 0.015 |  |
| Δ ROX 6 to 12 hours | | -2.04 (5.65) | -2.34 (6.78) | -1.94 (5.22) | 0.324 |  |
| Δ ROX 12 to 24 hours | | -1.4 (5.17) | -3.32 (5.41) | -0.89 (4.99) | <0.001 |  |
| Δ ROX greater a 24 hours | | -1.62 (5.85) | -4.11 (7.64) | -0.98 (5.17) | <0.001 |  |
|  |  | Total population n= 1402 | Death at 7 days n= 323 | Alive n= 1079 | p value |  |
|  |  |  |  |  |  |  |
| Δ PaO2/FiO2 ratio at 6 hours | | -25.21 (114.2) | -0.71 (93.87) | -26.91 (115.54) | <0.001 |  |
| Δ PaO2/FiO2 ratio 6 a 12 hours | | -35.83 (105.5) | -37.2 (79.18) | -35.74 (107.14) | 0.789 |  |
| Δ PaO2/FiO2 ratio 12 a 24 hours | | -8.38 (114.53) | -46.77 (121.16) | -5.69 (113.63) | <0.001 |  |
| Δ PaO2/FiO2 ratio greater 24 hours | | 174.52 (112.06) | 100.78 (108.08) | 177.32 (112.37) | <0.001 |  |
|  |  | Total population n= 1402 | Death at 14 days n= 323 | Alive n= 1079 | p value |  |
|  |  |  |  |  |  |  |
| Δ PaO2/FiO2 ratio at 6 hours | | -25.21 (114.2) | -44.8 (98.26) | -20.27 (117.7) | <0.001 |  |
| Δ PaO2/FiO2 ratio 6 a 12 hours | | -35.83 (105.5) | -52.03 (82.51) | -32.27 (109.77) | <0.001 |  |
| Δ PaO2/FiO2 ratio 12 a 24 hours | | -8.38 (114.53) | -21.88 (106.88) | -6.14 (115.42) | 0.023 |  |
| Δ PaO2/FiO2 ratio greater 24 hours | | 174.52 (112.06) | 107.97 (84.98) | 182.74 (115.18) | <0.001 |  |
|  |  | Total population n= 1402 | Death at 28 days n= 323 | Alive n= 1079 | p value |  |
|  |  |  |  |  |  |  |
| Δ PaO2/FiO2 ratio at 6 hours | | -25.21 (114.2) | -49.77 (103.91) | -12.71 (117.58) | <0.001 |  |
| Δ PaO2/FiO2 ratio 6 a 12 hours | | -35.83 (105.5) | -47.05 (99.24) | -31.25 (107.91) | <0.001 |  |
| Δ PaO2/FiO2 ratio 12 a 24 hours | | -8.38 (114.53) | -33.86 (118.82) | -1.01 (112.59) | <0.001 |  |
| Δ PaO2/FiO2 ratio greater 24 hours | | 174.52 (112.06) | 94.3 (83.68) | 180.38 (117.51) | <0.001 |  |

Notes: Δ: delta; SpO2/FiO2 ratio: arterial oxygen saturation in relation to the inspired oxygen fraction; ROX: Respiratory rate-OXygenation index; PaO2/FiO2 ratio: arterial oxygen pressure/inspired fraction of oxygen.
