# Supplementary material for: Performance of oxygenation indices and risk scores to predict invasive mechanical ventilation and mortality in COVID-19
Source: BMC Pulm Med. 2024 Feb 2;24:68. doi: 10.1186/s12890-023-02807-8 (PMC10835882; doi:10.1186/s12890-023-02807-8)
Supplement: Supplementary file 5 — Supplementary Material 5: Performance of oxygenation indices and risk scores in invasive mechanical ventilation and mortality at 7-14 days [file 12890_2023_2807_MOESM5_ESM.docx]

**Supplementary table 5.** Performance of oxygenation indices and risk scores in invasive mechanical ventilation and mortality at 7-14 days.

|  | S (IC 95%) | Sp(IC 95%) | PPV (CI 95%) | NPV (CI 95%) | LR+ (CI 95%) | LR- (CI 95%) | ROC(IC95%) |
| --- | --- | --- | --- | --- | --- | --- | --- |
| Invasive mechanical ventilation at 7 days | | | | | | | |
| PaO2/FiO2 ≤ 300 | 12.3 (10.6-14.1) | 71.9 (69.5-74.3) | 7.7 (6.3-9.1) | 81.2 (79.1-83.2) | 1.22 (0.847-1.757) | 0.44 (0.304-0.631) | 0.679 (0.634-0.724) |
| SpO2/FiO2 ≤ 350 | 51.9 (49.2-54.5) | 26 (23.6-28.3) | 11.4 (9.7-13.1) | 74.6 (72.3-76.9) | 1.85 (1.681-2.041) | 0.7 (0.613-0.801) | 0.658 (0.615-0.701) |
| ROX index ≥ 4.8 | 88.1 (86.4-89.8) | 2.7 (1.8-3.5) | 14.1 (12.3-16) | 55.4 (52.7-58) | 4.44 (4.216-4.666) | 0.91 (0.861-0.952) | 0.624 (0.579-0.67) |
| ΔPaO2/FiO2 ratio | 76.2 (73.9-78.5) | 6.7 (5.4-8) | 13.1 (11.3-14.9) | 60.3 (57.7-62.9) | 3.56 (3.295-3.845) | 0.82 (0.756-0.882) | 0.585 (0.538-0.631) |
| ΔSaO2/FiO2 ratio | 87.7 (85.9-89.6) | 7.5 (6-8.9) | 16.7 (14.6-18.7) | 74.3 (71.8-76.7) | 1.65 (1.56-1.735) | 0.95 (0.899-1) | 0.608 (0.565-0.65) |
| SOFA score ≥ 4 | 58.1 (54.6-61.7) | 75.9 (72.8-78.9) | 37.2 (33.8-40.7) | 88 (85.7-90.4) | 2.41 (1.978-2.933) | 0.55 (0.453-0.672) | 0.634 (0.593-0.675) |
| 4C score ≥ 8 | 74.9 (72-77.8) | 48.2 (44.9-51.5) | 25.3 (22.4-28.2) | 89.1 (87.1-91.2) | 1.45 (1.292-1.619) | 0.52 (0.466-0.584) | 0.649 (0.609-0.688) |
| Charlson index ≥ 3 | 68.5 (66.1-71) | 44.9 (42.3-47.5) | 18.7 (16.7-20.7) | 88.5 (86.8-90.2) | 1.24 (1.121-1.378) | 0.7 (0.633-0.778) | 0.557 (0.515-0.6) |
| Invasive mechanical ventilation at 14 days | | | | | | | |
| PaO2/FiO2 ≤ 300 | 13 (11.2-14.8) | 71.4 (69.1-73.8) | 9.7 (8.1-11.3) | 77.7 (75.5-79.9) | 1.22 (0.878-1.688) | 0.46 (0.329-0.633) | 0.683 (0.641-0.725) |
| SaO2/FiO2 ≤ 350 | 52.4 (49.7-55) | 25.8 (23.5-28.1) | 13.9 (12-15.7) | 70.3 (67.9-72.8) | 1.85 (1.635-2.035) | 0.71 (0.624-0.797) | 0.644 (0.602-0.685) |
| ROX index ≥ 4.8 | 88.9 (87.2-90.6) | 2.5 (1.7-3.3) | 17.1 (15.1-19.1) | 50 (47.3-52.7) | 4.42 (4.224-4.618) | 0.91 (0.872-0.953) | 0.622 (0.579-0.664) |
| ΔPaO2/FiO2 ratio | 88.1 (86.3-89.9) | 7.5 (6-8.9) | 19.9 (17.7-22.1) | 70.6 (68-73.1) | 1.6 (1.521-1.677) | 0.95 (0.907-1.003) | 0.597 (0.553-0.64) |
| ΔSaO2/FiO2 ratio | 88.5 (86.8-90.2) | 3.9 (2.9-5) | 17.5 (15.5-19.5) | 59.7 (57.1-62.3) | 2.93 (2.796-3.081) | 0.92 (0.88-0.965) | 0.602 (0.563-0.642) |
| SOFA score ≥ 4 | 55.9 (52.3-59.5) | 76.5 (73.5-79.5) | 41.1 (37.6-44.6) | 85.5 (83-88) | 2.38 (1.951-2.902) | 0.58 (0.473-0.703) | 0.634 (0.596-0.673) |
| 4C score ≥ 8 | 72.3 (69.4-75.4) | 48.5 (45.2-51.8) | 29.8 (26.7-32.8) | 86 (83.7-88.3) | 1.4 (1.247-1.579) | 0.57 (0.508-0.643) | 0.637 (0.599-0.675) |
| Charlson index ≥ 3 | 48.7 (46-51.3) | 61.4 (58.9-64) | 22.4 (20.2-24.6) | 84 (82-85.9) | 0.73 (1.082-1.472) | 0.84 (0.716-0.975) | 0.561 (0.521-0.6) |
| Mortality at 7 days | | | | | | | |
| PaO2/FiO2 ≤ 300 | 29.7 (27.3-32.1) | 74.7 (72.4-77) | 7.7 (6.3-9.1) | 93.7 (92.4-95) | 1.17 (0.843-1.63) | 0.94 (0.677-1.31) | 0.567 (0.482-0.652) |
| SaO2/FiO2 ≤ 350 | 62 (59.4-64.5) | 28.8 (26.4-31.2) | 5.9 (4.7-7.2) | 91.3 (89.8-92.8) | 1.32 (1.122-1.395) | 0.87 (0.738-1.025) | 0.567 (0.492-0.642) |
| ROX index ≥ 4.8 | 92.3 (90.9-93.7) | 3.8 (2.8-4.9) | 6.4 (5.1-7.7) | 87.5 (85.7-89.3) | 2 (0.904-1.02) | 0.96 (0.904-1.02) | 0.583 (0.503-0.664) |
| ΔPaO2/FiO2 ratio | 80.4 (78.3-82.6) | 8.6 (7.1-10.1) | 6.1 (4.8-7.3) | 85.7 (83.8-87.6) | 2.27 (2.051-2.516) | 0.88 (0.795-0.975) | 0.519 (0.444-0.594) |
| ΔSaO2/FiO2 ratio | 84.3 (82.2-86.3) | 7.9 (6.4-9.4) | 5.3 (4-6.5) | 89.2 (87.5-91) | 0.92 (0.826-1.014) | 1.98 (1.881-2.089) | 0.545 (0.471-0.618) |
| SOFA score ≥ 4 | 58.1 (54.6-61.7) | 75.9 (72.8-78.9) | 37.2 (33.8-40.7) | 88 (85.7-90.4) | 2.41 (1.978-2.933) | 0.55 (0.453-0.672) | 0.568 (0.499-0.638) |
| 4C score ≥ 8 | 74.9 (72-77.8) | 48.2 (44.9-51.5) | 25.3 (22.4-28.2) | 89.1 (87.1-91.2) | 1.45 (1.292-1.619) | 0.52 (0.466-0.584) | 0.666 (0.599-0.734) |
| Charlson index ≥ 3 | 49.8 (47.2-52.4) | 61.3 (58.7-63.8) | 19.2 (17.2-21.3) | 86.8 (85.1-88.6) | 1.29 (1.105-1.495) | 0.82 (0.705-0.953) | 0.608 (0.54-0.676) |
| Mortality at 14 days | | | | | | | |
| PaO2/FiO2 ≤ 300 | 22 (19.8-24.2) | 73.8 (71.4-76.1) | 13.1 (11.3-14.9) | 84 (82.1-86) | 1.06 (0.805-1.389) | 0.84 (0.638-1.102) | 0.627 (0.576-0.678) |
| SaO2/FiO2 ≤ 350 | 57.9 (55.3-60.5) | 27.1 (24.8-29.5) | 12.5 (10.8-14.3) | 78.1 (75.9-80.3) | 1.55 (1.376-1.752) | 0.79 (0.704-0.896) | 0.582 (0.533-0.632) |
| ROX index ≥ 4.8 | 88.9 (87.2-90.6) | 2.5 (1.7-3.3) | 17.1 (15.1-19.1) | 50 (47.3-52.7) | 4.42 (4.224-4.618) | 0.91 (0.872-0.953) | 0.588 (0.537-0.64) |
| ΔPaO2/FiO2 ratio | 78.5 (76.3-80.7) | 7.1 (5.7-21.5) | 13.4 (11.6-15.3) | 64.3 (61.7-66.8) | 3.02 (2.81-3.251) | 0.84 (0.786-0.909) | 0.522 (0.476-0.569) |
| ΔSaO2/FiO2 ratio | 89.6 (87.8-91.3) | 8 (6.5-9.5) | 14.6 (12.6-16.6) | 81.4 (79.2-83.6) | 1.3 (1.236-1.374) | 0.97 (0.924-1.026) | 0.59 (0.542-0.638) |
| SOFA score ≥ 4 | 58.1 (54.6-61.7) | 75.9 (72.8-78.9) | 37.2 (33.8-40.7) | 88 (85.7-90.4) | 2.41 (1.978-2.933) | 0.55 (0.453-0.672) | 0.62 (0.576-0.664) |
| 4C score ≥ 8 | 74.9 (72-77.8) | 48.2 (44.9-51.5) | 25.3 (22.4-28.2) | 89.1 (87.1-91.2) | 1.45 (1.292-1.619) | 0.52 (0.466-0.584) | 0.714 (0.671-0.756) |
| Charlson index ≥ 3 | 49.8 (47.2-52.4) | 61.3 (58.7-63.8) | 19.2 (17.2-21.3) | 86.8 (85.1-88.6) | 1.29 (1.105-1.495) | 0.82 (0.705-0.953) | 0.675 (0.632-0.717) |

Notes: S: Sensibility; Sp: specificity; PPV: positive predictive value; NPV: negative predictive value; LR +: positive likelihood ratio; LR-: negative likelihood ratio; CI: confidence intervals; ROC: receiver operating characteristic curve; PaO2: arterial oxygen pressure; SaO2: arterial oxygen saturation; Δ: delta; PaO2/FiO2 ratio: arterial oxygen pressure/inspired fraction of oxygen; SaO2/FiO2 ratio: arterial oxygen saturation in relation to the inspired oxygen fraction; ROX: Respiratory rate-OXygenation index; SOFA: Sequential Organ Failure Assessment.
